# Let's put a person back into Cyber-Physical-Social research: Public Mental Models Framework

**DOI:** 10.3389/fnrgo.2025.1520434

**Published:** 2025-11-26

**Authors:** Mare Teichmann, Jaanus Kaugerand, Merik Meriste, Kalev Rannat

**Affiliations:** Laboratory for Proactive Technologies, Department of Software Science, Tallinn University of Technology, Tallinn, Estonia

**Keywords:** mental models, levels of mental models, situation awareness, Public Behavior Patterns, Public Mental Models, Public Mental Models Framework

## Abstract

In the current paper our focus is on linking Public Mental Models with behavior, Situation Awareness and stress management, with predicting and intervening in public behavior in critical situations. Understanding and influencing behavior within complex Cyber-Physical-Social Systems (CPSS) requires an explicit link between mental models, behavior, situation awareness, and stress management. This paper introduces the Public Mental Models Framework (PMMF) as a systematic approach for analyzing and predicting public behavior in critical situations, thereby improving adaptive decision-making and person—AI collaboration. The PMMF explains how internal and external indicators such as cognitive, social, cultural, political, economic, and technological, that shape perception and behavioral responses across multiple levels: individual, team, organizational, community, and societal. By identifying these triggers and markers, the framework supports why behaviors deviate or stabilize under stress, providing an analytical basis for targeted interventions and resilience-oriented design. In contrast to traditional Situation Awareness models that emphasize what is perceived and how it is processed, PMMF focuses on the interpretive mechanisms through which actors construct meaning and make decisions. Integrating PMMF with the Motivation-Opportunity-Ability (MOA) theory enables systematic assessment of behavioral potential and performance within CPSS. This integration strengthens the neuroergonomic foundation for evaluating human and AI entities and enhances the capacity to design interventions that foster informed, adaptive, and ethically aligned behavior in complex sociotechnical environments.

## 1 Introduction: Public Mental Model perspective

The existing gap that the Public Mental Models Framework (PMMF) fills beyond traditional Situational Awareness (SA) and Mental Model (MM) frameworks is that it explicitly extends analysis from the individual and team cognitive levels (covered by MM and SA) to the societal or public level, focusing on how shared mental constructs emerge, evolve, and influence collective behavior within Cyber-Physical-Social Systems (CPSS). In other words: SA deals primarily with how individuals or teams perceive and comprehend a situation for decision-making; MM frameworks explain how individuals internally represent and interpret reality; PMMF, however, addresses the missing link between these—namely, how multiple individual mental models converge into Public Mental Models (PMMs) that shape Public Behavior Patterns (PBPs) across society, especially under stress or crisis. For example, PMMF could support both predictive behavioral modeling for crisis management and trust-aware system design in hybrid human—AI environments, making it a bridge between cognitive science, AI, and societal resilience.

In an era of rapidly evolving technological, social, and environmental challenges, understanding how individuals and societies interpret, react to, and make decisions in complex situations is of critical importance. The Public Mental Model Framework proposed in current paper builds on our previous works, where we have focused on critical situations including crisis management through the lens of situational awareness (SA) and Cyber-Physical-Social Systems (CPSS). This paper explores the role of Public Mental Models (PMMs) in critical situations. In this context, the term “public” refers to collective or societal-level cognition, how shared mental models emerge within a population, it also can be expressed as public opinion, but it is not in the meaning of governmental or civic institutions. We seek to expand the conceptual foundation for anticipating, interpreting, and guiding public behavior by analyzing how individual mental models (MMs) evolve and coalesce into collective cognitive representations, or Public Mental Models (PMMs). Mental Models (Gray et al., 2013) serve as cognitive tools (Peperkorn et al., 2024) that filter information, shape expectations, and direct actions. When shared, these models create PMMs which emerge from collective cognition within social contexts, influencing public behavior and responses during critical situations and crises. In such way the PMMF could provide a useful societal-level perspective. However, it does not currently delve into the specific cognitive or communicative mechanisms by which individual mental structures are transformed and integrated into collective representations. This omission leaves open important questions about how shared understandings emerge from individual cognition, how meaning is negotiated within groups, and how these processes are shaped by context and interpersonal dynamics. To address this limitation, our framework is informed by classical group dynamics theories from psychology and sociology—particularly those concerning norm formation, social identity, group cohesion, and collective decision-making. These theories provide a robust foundation for understanding how individuals contribute to, and are shaped by, shared mental constructs in both stable and high-stress environments. The PMM framework proposed relies on the concept of Public Behavior Patterns (PBP) (Teichmann et al., 2022), which delineate recurring actions of individuals and groups in public settings and introduces the role of PMM in shaping these behaviors. In CPSS—comprising smart cities, autonomous systems etc.—where understanding PMMs is critical for real-time system adaptation and decision-making, the advanced AI and machine learning (ML) applications could allow insights into Public Behavior Patterns (PBP). The PBP can also involve person—AI collaboration and cases of hybrid intelligence (Petrescu and Krishen, 2023). In these cases the behavior patterns of the people are intertwined and influenced by technology e.g., situation aware systems (D'Aniello et al., 2022). For example, AI and LLM based assistants such as Google Gemini, OpenAI's GPT-4, Meta's LLaMa, Apple Siri, Amazon Alexa, etc. The question is how we can better understand and enhance the behavior and performance of complex CPSS through the integration of mental models, human-AI collaboration and hybrid intelligence. It should be also mentioned that AI and large language based models only mimic the human cognitive processes by responding based on learned patterns from data rather than possessing a framework for understanding societal-level Public Mental Models when in interaction with humans (Harel and Marron, 2024). AI models are trained to generate contextually relevant and coherent responses. However, these systems do not use the concept of a PMM but rely on the (Large Language Models) LLM (text based, language structures) data they were trained on and the neural network architecture that enables pattern recognition and language generation. In this way the data collection does not inherently build or refine a Public Mental Model (PMM). In AI and machine learning, the concept of PMM is not a standard term.

Our research draws on three primary approaches: (1) Mental Models focus on individual cognitive processes; (2) Situation Awareness (SA), where MM supports SA for improved decision-making; and (3) Behavioral Analysis, studying public actions as reflections of shared cognitive models. The proposed Public Mental Models Framework (PMMF) integrates these approaches, enabling proactive, human-centered CPSS design and management. Inspired by neuroergonomics, AI, and digital twins, PMMF offers a means to simulate, predict, and influence PBPs, aiding in the anticipation of public reactions to critical events and enabling adaptive crisis response strategies. The framework also considers the need for ongoing refinement, as PMMs are constantly influenced by social domains like culture, politics, and technology. Our previous research highlighted that stress and situational awareness significantly impact crisis response, supporting the link between public satisfaction, infection rates, and PMM-informed behavior (Teichmann and Jakobson, 2021). In CPSS, integrating a human-centered approach requires early incorporation of public behavior insights and social indicators. However, despite looking very promising, designing an effective Public Mental Models Framework that allows to plan strategies to intervene in public behavior within the CPSS context requires careful conceptualization. The dynamic and evolving nature of PMMs necessitates continuous refinement of the PMMF, as PBP is influenced by markers, triggers, and drivers from social domains like culture, cyber, medicine, politics, and economics.

## 2 Levels of mental models

**Figure 1:**
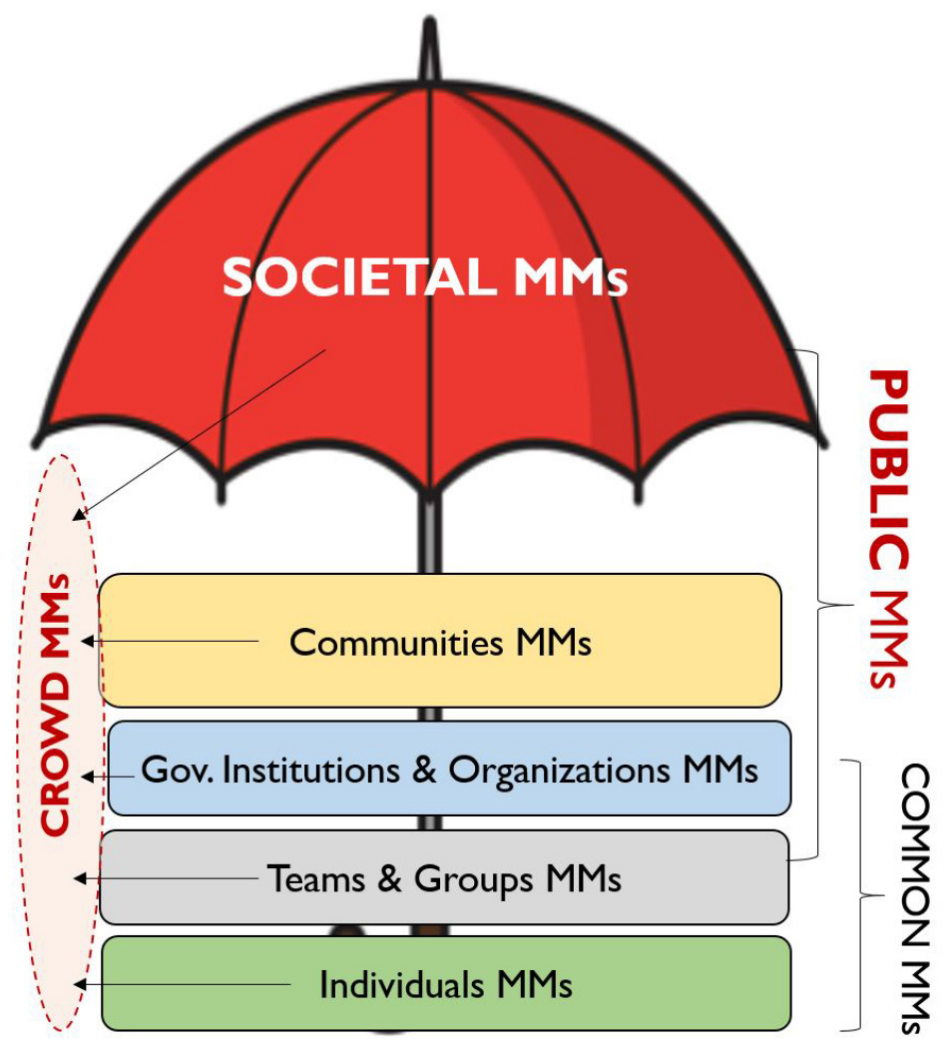
Levels of mental models.

In neuroergonomics, the categorization of mental models at different societal levels (e.g., individuals, teams, organizations, communities, and societies) mirrors the interdisciplinary approach, see Figure 1

We categorize mental models into the following levels: (1) individual level—individual mental model; (2) team or group level—common mental model; (3) organizational level—multiple individuals, teams, or groups—organizational mental models; (4) community level—communities' mental models; (5) society level—societal mental models. Additionally, we attempt to define these different levels of mental models with the focus of public and common mental models as depicted on Figure 1. This interdisciplinary classification helps to understand the interactions, influence, and shared mental models that exist between individuals, groups, organizations, and entire societies. It is similar to classifications found in sociology, psychology, organizational studies (Rapp et al., 2022; Atweh et al., 2022).

Considering mental models at different levels help in critical situations to better understand, predict and manage behavior patterns that can be anticipated in communities and crowds. Individuals' mental models are personal tools each person uses to understand, interpret, and interact with the world. These models are shaped by personal experience, education, culture, and individual biases. Examples include a person's approach to problem-solving, views on leadership, or understanding of market dynamics. Team or group level mental models are shared among small groups of people, such as a project team or department within an organization. Members of these teams or groups develop shared mental models to coordinate their actions and collaborate effectively. At the level of governmental institutions and other type of organizations, shared mental models exist across entire organizations. Mental models reflect the organization's culture, values, and strategies, as well as the collective ways people within the organization interpret events and make decisions. Examples include a company's perspective on competition or customer service, innovation practices within a ministry, or local council. Community level mental models are shared among communities—groups larger than teams or organizations but smaller than societies, often bound by shared values, geography, or purpose. Models are shaped by shared experiences and social norms. Examples include neighborhood communities, cultural and ethnic communities, religious groups, interest-based communities, professional associations, social identity communities, political groups, and online communities. At the societal level, mental models are widespread across a nation or global society. This level encompasses broad-scale dynamics that affect entire societies. These mental models represent widely shared beliefs or understandings within a society, including culture, social norms, public opinion, and mass communication. Examples include public opinion on climate change, democracy, technological progress, attitudes toward public health systems, or societal movements. In this context PMM refers to the collective understanding or cognitive framework held by a large group of people, typically on a societal or public scale phenomena such as politics, public policy, environmental issues, etc. There can be many Public Mental Models coexisting at the same time within the society. A public mental model can be varied, representing a spectrum of viewpoints but with identifiable dominant trends or patterns i.e., shaped by factors like media, culture, education, politics, economy and shared experiences. For example: a public mental model of climate change or economic development or something else that is currently an important issue in society is shared by many, while the others may be skeptical and share another PMM of economic development or attitude toward, for example, abortion. Meaning, each PMM formed within a large group of people needs to be based on some shared context, experience, common ground or event. It is also possible that an aggregate of MM of a social group that is forming consists only of individuals with MMs with no alignment or common ground at all (Teichmann and Motus, 2024). Common mental models, on the other hand, are more specific and typically apply to smaller, goal-oriented groups (e.g., teams), where there is a stronger alignment of objectives and knowledge (teams, organizations, professional communities). In this context common MM include tasks, goals, or projects requiring cooperation, such as engineering teams, business strategy groups, or even military operations. For example: common mental model exists within an engineering team about how a particular project should be developed, where all team members share the similar vision and approach. Formation of both PMMs and CMMs for a given situation are significantly influenced by contextual conditions and the emergence of ad-hoc groups and their interactions. Both PMM and CMM comprise a network of beliefs and attitudes about how the world works in a certain situation, rather than being rooted in factual truths. It's important to note that the group of people who accept a PMM or CMM are aware of and expect this shared understanding, even if they can't articulate it verbally or mentally.

## 3 Mental models approach

An individual uses mental models to comprehend the functioning of the world. For any activity in a particular situation, mental models direct our perception and attention during the process of gathering information. Mental models then aid in the interpretation and comprehension of the gathered information, allowing us to assess the situation. Mental models help reasoning and decision-making processes, thereby influencing a person's actions. In general, any application of available particular mental models and the creation and refinement of new ones are influenced by both internal and external factors. Internal factors pertain to the specific individual and include aspects such as fatigue, perception, attention, memory capacity, experience, knowledge, motivation, and stress. External factors, on the other hand, are mostly independent of the individual and encompass various elements originating from the socio-cultural environment, which is formed by domains of culture, cyber, politics, social, economic, and varies by time and place. A public mental model (PMM) emerges when a community of interacting individuals (i.e., the public) share their mental models regarding a specific situation or circumstance. External factors impact the formation of a PMM, as individuals with similar backgrounds collectively and unintentionally develop a shared mental model. A PMM is also significantly influenced by contextual conditions, including the composition of ad-hoc groups, their interactions, and the underlying reasons for those interactions, all of which contribute to the shaping of a PMM related to a particular situation. A PMM is a network of interconnected public beliefs and attitudes concerning the functioning of the world or a specific situation. Individual mental models are dynamic, they undergo a gradual process of change and refinement as individuals learn new things about the world. However, under normal circumstances, mental models tend not to change rapidly. While a person's mental models might evolve in certain areas as they acquire new skills and experiences, significant changes are infrequent.

The ongoing debate regarding the meaning and definition of Public Mental Models (PMMs) has led to the dominance of the Mental Models approach in PMM literature. This approach views Mental Models as cognitive structures unique to individuals that enable them to structure, interpret, and make sense of the world around them. The Mental Models approach simplistically regards a PMM as a collection of distinct Mental Models held by various individuals in the same situation. According to this approach, the concept of Mental Models represents an understanding of how something operates and/or interacts with the real world, arising from complex human cognitive processes (Cárdenas-Figueroa and Navarro, 1943). Evidence-based Mental Models permit us to draw conclusions, validate them in real-world scenarios, simulate potential future situations, and make informed decisions when addressing real problems. A Mental Model encompasses the individual's comprehension of the surrounding world, the relationships among its components, and their intuitive perception of their actions and the resulting outcomes (Zwaan, 2016). Individuals formulate Mental Models in their minds to comprehend how things operate, approach challenges, and make decisions (Craik, 2012; Santamaría et al., 2013). Mental Models assist people in interpreting their environment and making sense of the world. Previous knowledge and experience shape Mental Models, which can be refined through feedback and reflection. They serve as psychological representations of real, hypothetical, or imaginary phenomena or situations, playing a pivotal role in decision-making and actions. Mental Models are the outcome of perception and comprehension, serving as the foundation of reasoning (Rook, 2013). In the academic literature, the content of Mental Models are described as rational and/or intuitive generalizations, representing someone's cognitive processes concerning the operation of something in the real world (Lindstrom, 2010; Dietrich et al., 2013). They can also manifest as collections of ideas, opinions, and assumptions, including trust in something or someone (Jordaan, 2019). Mental Models can take the form of networks of facts and concepts (Villareale et al., 2022; Zhang et al., 2022), or cause-and-effect systems (maps) and schemas, scripts (Keller et al., 2021; Hochpöchler et al., 2013). In this context, Mental Models can be described as cognitive structures within our minds (resembling gestalt and schemas) that individuals use to organize and interpret information. Although each person's Mental Models are unique, certain combinations of Mental Models can become widely shared and accepted by the public or society, resulting in the emergence of a Public Mental Model (PMM). Based on the previously described scientific positions, our understanding is that the process through which a set of Mental Models transforms into a PMM may involve the following steps: (1) Information based on individuals' distinct Mental Models is exchanged through interactions, evolving into a shared understanding. These interactions can occur through direct communication between holders of Mental Models, indirect interactions through the environment, or mediation via various communication channels, such as different forms of mass and social media. (2) Formation of a shared common understanding: Mental Models become a PMM when a shared understanding develops among a group of individuals or within a society. This can be facilitated through communication, education, or shared experiences. (3) Validation and integration into culture: A widely accepted and validated PMM can become ingrained in the culture of a society, potentially being reflected in art, literature, and other cultural artifacts.

## 4 Situation awareness approach

Mental models (MM) are integral components in explaining and achieving situation awareness (SA), which is essential for decision-making and performance in complex, dynamic environments. Erroneous mental models can lead to poor SA and subsequent failure in tasks (Jones and Endsley, 1996). This section reviews SA and MM at both individual and system levels. SA, a concept that gained traction in the late 1980s in the field of ergonomics, has applications in fields such as aviation, military, cybersecurity, and emergency response (Gorman et al., 2017; Nguyen et al., 2019; Oliveira et al., 2019). Despite its widespread use, a unified definition remains elusive. Endsley's model (Endsley, 1995) is by far the most widespread, but alternative approaches such as situated SA (Chiappe et al., 2020), activity-theory based SA (Bedny and Meister, 1999), adaptive externally directed consciousness (Smith and Hancock, 1995), sensemaking (Klein et al., 2007), comprehension SA (Adams et al., 1995) have also been proposed. The spread of the ideas of distributed cognition theory and systems thinking have led to a view that SA might be a system level phenomenon as noted in the works of Artman and Garbis (Artman and Garbis, 1998, Aug 24–16), Chiappe, Strybel and Vu (Chiappe et al., 2020) and Stanton et al. (Stanton et al., 2006). It is therefore not agreed upon whether SA is something an individual possesses or whether it is something that emerges as a system level phenomenon through interactions between an individual and the environment.

According to Endsley, SA is the “... ongoing state of knowledge necessary for decision-making in complex and dynamic domains.” (Endsley, 2021). SA is not the whole set of knowledge one possesses but only knowledge related to the current situation and in support of one's goals. This knowledge is maintained in long-term memory in the form of schemata and mental models. More precisely Endsley (Endsley, 1995), using the ideas of Mayer (Mayer, 1992), describes how the perceived information is coded and stored in long-term memory as more abstract schemata describing different prototypical situations. Schemata represent a person's general knowledge about particular situations, including among others declarative knowledge (i.e., facts) and procedural knowledge (i.e., how something functions). Schemata are learned and continuously refined through experience. Mental models, as described by Rouse and Morris, enable individuals to understand system functioning and predict future states (Rouse and Morris, 1986). These models help circumvent the limitations of working memory by enabling efficient information processing, comprehension, and projection (Endsley, 2015). Distributed situation awareness (DSA) expands on this by viewing SA as an emergent property at the system level. In the DSA model proposed by Stanton et al. (2006); Salmon and Plant (2022), SA is a collective property influenced by interactions among system agents, who process and share information based on their individual tasks and goals. Transactions between agents ensure that their SA remains compatible, allowing the system to function effectively (Stanton et al., 2017). Alternative approaches to SA include Bedny and Meister's application of Russian activity theory (Bedny and Meister, 1999), which views SA as part of a broader reflective-orientational activity. In their model, SA emerges during the process of creating subjective models of reality, where goals and motivation are key. Similarly, Smith and Hancock (1995) define SA as adaptive, externally directed consciousness, emerging as operators cycle through evaluating and modifying their schemata. Other models align with either Endsley's or Stanton's frameworks. Sarter and Woods (1991) describe SA as all accessible knowledge that can be integrated to form a coherent picture, noting that adequate mental models are essential for SA. Maintaining SA imposes a high cognitive load in complex situations and operators manage the currently active schema by keeping in working memory only tokens of larger knowledge structures in long term memory (Adams et al., 1995). Durso and Gronlund (1999) also support idea that that working memory holds pointers to long-term memory structures, while Chiappe et al. (2020) propose a situated approach to SA, where operators also rely on external environmental cues by also offloading information onto the environment and access it when needed (a step towards distributed cognition). At the team and organizational levels, SA becomes a shared, distributed phenomenon (Stanton et al., 2017). Team SA emerges from the integration of individual members' awareness, each shaped by personal experience, goals, and mental models (Hornsey et al., 2023). Compatible awareness across team members is essential for maintaining cohesion and effective functioning in distributed systems (Stanton et al., 2017). In organizations, this shared awareness spans multiple levels—individual, group, organizational, and public (Hornsey et al., 2023).

## 5 Behavioral approach

The Behavioral Approach to Public Mental Models (PMM) represents a combined perspective on PMM and emphasizes how public behavior and interactions reveal mental models. Within this framework, the focus is directed towards public behavior—what individuals actually DO and how they interact. People's behavior and interactions are regarded as “tools” for behavior modelling to uncover and derive conclusions about their mental models (Hall, 2011). This also applies to Public Behavior—generalized inferences can be drawn about the behavior patterns of social groups of individuals. For instance, data from social surveys or cyber-physical social systems (CPSS), such as situation aware wearable technology that tracks environmental cues and adapts to users' behaviors, can help understand how mental models and situation awareness are interconnected (D'Aniello et al., 2022). The relationship between behavior, situation awareness, and mental models, and how shared versions of mental models and situation awareness are fostered through interactions, is essential in the study of Public Mental Models. Shared mental models may lead to aligned situation awareness and comparable behavior, and vice versa, whereas diverse mental models could result in intricate interactions and the emergence of collective-public behavior that is highly challenging to predict. This understanding assists in exploring different conceptualizations and modelling approaches of PMM, and their resilience in the face of prolonged crisis situations. Academic literature offers numerous examples of exploring the role of Mental Models in individual behavior. For instance, Mental Models play a pivotal role in how individuals respond to and cope with unexpected or critical situations. Gigerenzer et al. (1991) have extensively explored how mental models shape public comprehension of risk and uncertainty. They have also posited that Public Mental Models can be enhanced through improved communication and education. Tversky and Kahneman (1974) delved into how Public Mental Models influence judgments and decision-making in uncertain scenarios. By gaining a better understanding of how PMM influence people's behaviors and coping mechanisms during crises, individuals and practitioners can work towards developing more adaptive behavior and coping strategies that foster resilience and recovery. Firstly, PMM can impact how perception, interpretation, and understanding evolves in a critical situation. For instance, an individual with a PMM that perceives the world as perilous and unpredictable might be more inclined to view the crisis as a significant threat, leading to heightened anxiety and stress (Ho et al., 2022). Secondly, appropriate PMM could help to predict behavior, meaning that it can shape how individuals and groups make decisions and determine their course of action during a crisis. For example, an individual with PMMs that value self-reliance and independence may be more likely to take steps to protect themselves and their loved ones, even if that means diverging from official guidance or societal norms (Berg et al., 2022). Thirdly, PMM can also influence the coping strategies that individuals adopt to manage the stress and uncertainty of a crisis. For example, an individual with a PMM that values problem-solving and action may be more inclined to seek out information and take measures to address the crisis, while someone lacking these values might withdraw and disengage (Berlin and Adams, 2017). Fourthly, a better understanding of PMM during crises is essential because it aids in predicting the behavior patterns of individuals and groups. This helps the planning of future actions by rescuers, enhances communication with affected individuals and groups, prepares for providing understandable instructions and clear guidelines for crisis behaviors, engages affected individuals and groups, develops more effective operating instructions for rescuers, and mitigates human suffering and crisis-related consequences. Drawing from the aforementioned examples, it is plausible to arrive at generalization about the PMM of a social group of individuals.

## 6 Toward Public Mental Models Framework

In the following sections, we explain our reasons and background relevant for the developing conceptual Public Mental Models Framework (PMMF). It must be stressed that the framework is presented at conceptual level, the wide range of possible situations and computational problems require rethinking of fundamentals and conceptions. To provide context for the following chapter, we consider critical situations, and situation management. Although critical situations are often straightforward in terms of providing a source of common ground, shared beliefs, and collective understanding for respective and possible also different Public Mental Models (PMM) to form, they also present uncertain and information-poor scenarios, making decision-making difficult. In our previous works we focused on comprehensive framework for crisis management from SA and CPSS point of view. In this paper we focus linking Public Behavior Patterns (PBP) to PMM. Cyber-Physical-Social Systems (CPSS) ties into the neuroergonomics and focus on designing human-centered systems that account for human cognitive capacities. For example, consider a pandemic communication scenario where uncertainty and stress rapidly alter public perception. Health authorities, guided by situational awareness and behavioral data, could anticipate how evolving Public Mental Models (PMMs) shape trust in institutions, compliance with regulations, and acceptance of preventive measures. By applying the PMMF, one could identify key societal markers: such as trust in expertise, perceived personal control, or fatigue with restrictions, that drive Public Behavior Patterns (PBPs). This allows designing adaptive communication strategies or interventions that strengthen motivation and ability while reducing stress-induced misinformation spread. A similar PMMF-based approach could support AI trust modeling, where public confidence in autonomous systems evolves through shared beliefs and collective experience.

CPSS should integrate firstly human and then, technological, and physical elements, requiring an understanding of how users interact with technology and how cognitive factors like stress, situational awareness, and mental models influence system use. Firstly, we identified six core psychological concepts—Fitting Dilemma, Trust, Evidence-Based Situation Management (SM), Control vs. Locus of Control, Risk Perception and Awareness, Performance and Competence, and Stress—that are beneficial for integrating into a more comprehensive framework for crisis management (Teichmann and Jakobson, 2023). Secondly, from a Comprehensive Situation Awareness (CSA) and CPSS modeling perspective, we discussed some of the challenges encountered during the design of a system aimed at creating comprehensive situational awareness for a small country (Motus et al., 2019). This work aimed to merge the experience gained from modeling and analyzing Cyber-Physical-Social Systems with the results published on Situation Awareness. To this end, we developed a simple and efficient monitoring framework that captured and analyzed dynamic phenomena in a country. The ultimate goal was to build a model for comprehensive situational awareness that integrates information from a feasible set of interoperable models, which describe the functioning of a country's major institutions, and provides situational insights for decision-makers at all levels. Additionally, we explored two frameworks, one for efficient monitoring to capture and analyze heterogeneous dynamic phenomena in a country, and the second to model Comprehensive Situation Awareness (Teichmann and Motus, 2024). This latter framework included also competence variables (from MOA theory) and integrated key variables for cognitive processes based on psychological theories. In the context of neuroergonomics, it is crucial to estimate the performance of entities within Cyber-Physical-Social Systems. We conclude that to achieve this it looks promising to integrate the suggested PMMF approach with motivation-opportunity-ability (MOA) theory or similar approaches (Michie et al., 2011). The MOA Theory posits that performance and behavior are driven by three main factors:

- Motivation: The desire or willingness to perform a behavior.
- Opportunity: The environmental or contextual factors that facilitate or hinder performance.
- Ability: The skills or competencies required to perform the behavior.

By linking PMMF approach with MOA theory, we keep focus on public behavior and estimating the performance of social aspect in Cyber-Physical-Social Systems. For example, the PMMF identifies through the collection and analysis of information about societal markers that can influence motivation and external opportunities, such as political and cyber markers can either create opportunities or barriers for public engagement and considers ability factors within public behavior patterns. Enhancing ability involves providing the necessary skills or resources through targeted interventions, like educational programs or digital literacy campaigns, which align with the markers identified as influential. Also, we have found that the existing literature did not sufficiently address the relevance of Common Mental Models (CMM) at organizational level (Teichmann and Motus, 2021). We found empirical evidence related to changes in job satisfaction determinants before and during the COVID-19 pandemic, while at the same time the stress at work was significantly increased. The most intriguing finding was the statistically significant increase in the satisfaction with work environment, which became during pandemic to work predominantly from home, and it was also significantly related to job satisfaction improvement.

In current paper we argue that since PMM serves as the foundation for public understanding, behavior, and decision-making, the most effective way to model and to guide public behavior could be to address its Public Mental Models (not solely situational awareness, but also psychological factors). For example, in our previous work (Rebane et al., 2022), we focused on the relationship between public satisfaction with crisis management and the pandemic's progression, based on the Estonian case. We analyzed data from 37 COVID-19 survey reports conducted by the Estonian State Office and collected weekly data on new COVID-19 cases in Estonia from the World Health Organization (WHO) (Teichmann et al., 2022). Our analysis showed a strong negative correlation between public satisfaction with crises management and infection rates—lower satisfaction with crises management coincided with higher infection rates, and vice versa. This indicates the existence of a PMM related to public satisfaction with crises management. Therefore, we proposed the concept of Public Behavior Patterns (PBP) to describe recurring public behaviors patterns (PBP) during crises (Teichmann et al., 2022). The paper demonstrated, based on statistical surveys, that a clear connection exists between PBP and cultural background, influencing behavior. Also, we have analyzed the negative impact of high stress levels on an individual's cognitive situational awareness and situation management capabilities. This is a critical aspect to consider when designing people-centered Cyber-Physical-Social Systems (CPSS). High stress levels can impact the ability to perceive and interpret information accurately, leading to inadequate situational awareness and poor decision-making. This aspect of how stress can also make it difficult for people to follow rules and regulations in crises was highlighted by Teichmann and Jakobson (2021). Accordingly, SA can be influenced by high-stress situations, where cognitive overload diminishes an individual's ability to make sense of complex information. High stress levels and cognitive overload impairing an individual's ability to process information, leading to diminished situational awareness and poor decision-making is important understanding. Also, in neuroergonomic, these factors are studied to develop systems that reduce cognitive load and enhance performance, especially in environments where human operators (e.g., healthcare workers, first responders) are likely to experience the high levels of stress and cognitive overload. However, accurate Public Mental Models and continuous monitoring can enhance SA, allowing for better public responses. The previous studies on situational awareness (SA) suggest that mental models (MM) may play a central role in determining it (Endsley, 2015). Even though mental models are cognitive representations of external reality and tend to be functional rather than complete or accurate, people use these simplified representations to interact with the real world (Teichmann and Motus, 2024).

The proposed PMMF grows out from and relates to our previous works and its aim is to improve both our understanding of how to comprehend and influence the behavior of social groups or crowds, especially during prolonged crises. It is a conceptual work in progress and this paper presents our initial vision of it. The PMMF aims to lay the foundation for designing digital twin-type models to predict and intervene in Public Behavior Patterns (PBP). PMMF creates a virtual environment for constructing digital representations of Public Mental Models, conceptualizing how public behaviors are shaped by shared beliefs and cognitive processes. In addition, AI solutions aiding or collaborating with humans could make use of this framework to understand how humans or the public perceive the world or specific situations (Floridi, 2025).

**Figure 2:**
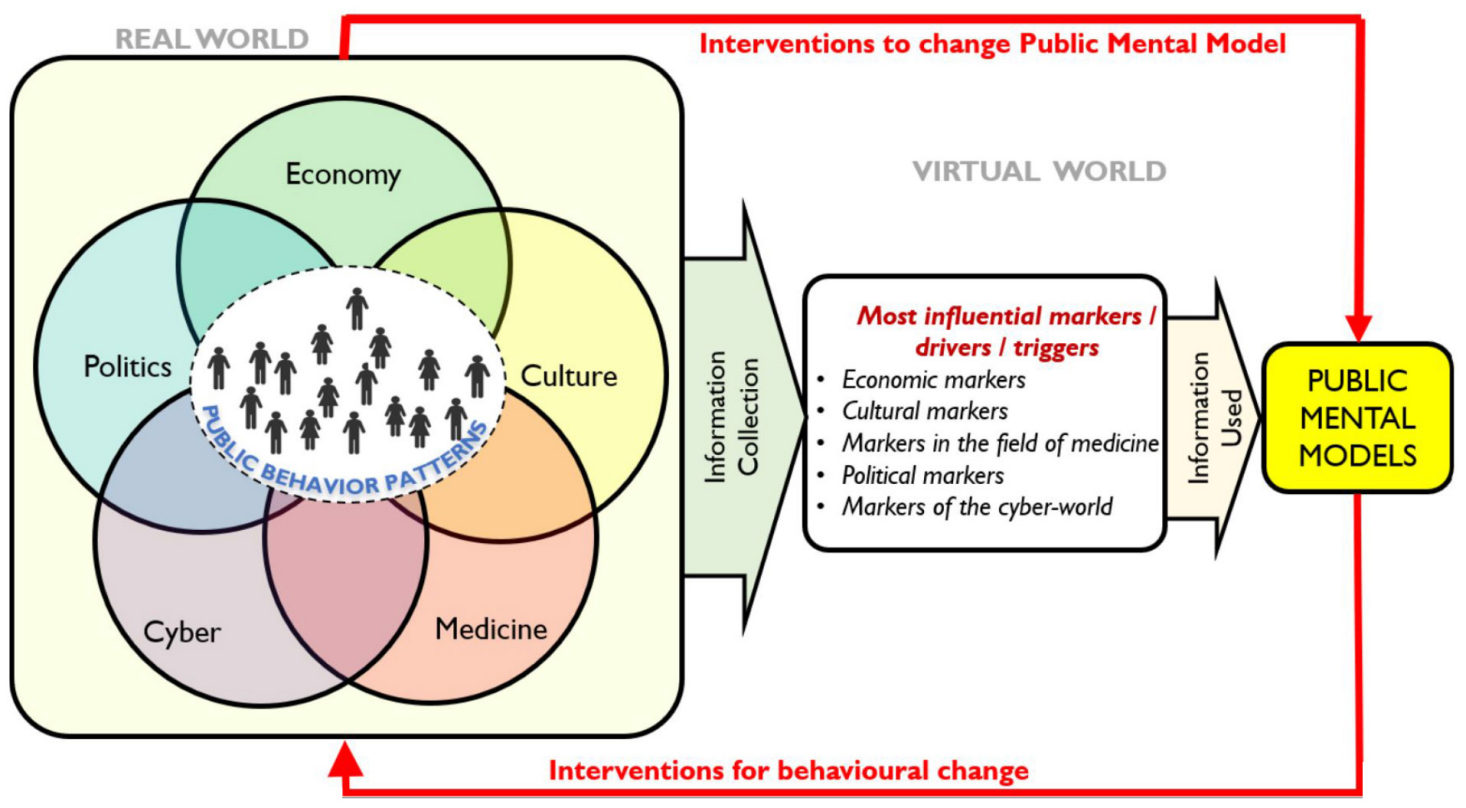
Public Mental Models Framework.

As neuroergonomics similarly focuses on understanding human factors to design systems that account for how people behave and make decisions in various contexts, we strongly see, that it can offer valuable insights and methods to design digital representations that are responsive and aligned with human cognitive and mental models, making also human-technology interaction more intuitive and efficient. By our interpretation the PMMF aims to help predicting and guiding collective behavior during crises. Neuroergonomics similarly focuses on understanding human factors to design systems that account for how people behave and make decisions in various contexts, particularly during critical incidents where public safety is a concern (e.g., large crowds in crises). The PMMF highlights the relationship between PMM and PBP, as shown in Figure 2. It consists of two key components:

1. The Real World: Where public behavior patterns emerge and are observed and tracked.
2. The Virtual World (VW): Where data is collected (e.g., surveys, social media analysis) and used to analyze and simulate Public Mental Models for behavior prediction.

Public Mental Models are influenced by various societal domains, including economy, culture, medicine, technology, and politics. The first step in analyzing these patterns in the real world and constructing PMM representations involves gathering behavioral and contextual data from trustworthy sources such as public opinion surveys, cultural trends, economic conditions, academic research etc., but also from selected parts of social media and news articles. From this data specific indicators like markers, drivers, and triggers, that have the most impact on public behavior and mental models, can be selected:

- Markers: Data points, events, or trends that provide insight into public responses and behaviors. For example, economic markers might include unemployment rates, cultural markers might involve prevailing social norms, political markers could be recent policy changes, etc.
- Triggers: Specific events or circumstances that cause shifts in public behavior, like disasters, public health crises, technological changes, or political events.
- Drivers: The underlying factors shaping public behavior, such as socioeconomic conditions, cultural norms, technological developments, and political decisions.

The inference and analysis of these influential indicators based on advanced AI analytics (including natural language processing) provides a broad and nuanced picture of the current environment (situation) that reflects a wide array of influences on public behavior—ranging from economic fluctuations and cultural shifts to political developments, cybersecurity threats, and health data. For instance, a sudden spike in unemployment might highlight an economic strain, while a viral misinformation campaign could signal emerging issues within the digital realm. These markers serve as early signals of significant shifts in public sentiment or behavior. As the second step the critical task is of making sense of how these indicators (i.e., markers, triggers, drivers) complement with each other to shape public behavior. Advanced AI, Machine Learning (ML) or statistics-based analytics could be applied to understand and extract patterns of public behavior. During this analysis the algorithms could cluster similar behaviors in context with thoughts, beliefs, and perceptions, to synthesize a digital representation of Public Mental Models to capture collective beliefs and attitudes, shaping how the public perceives and responds to different situations. However, we also acknowledge that these indicators can be imprecise and uncertain, leading authors to suggest including a formal method such as fuzzy logic to analyze their influence and role in formation of PMMs.

Fuzzy logic allows dealing with imprecise and uncertain information, which is inherent in human perception and decision-making processes. In the context of PMMs, fuzzy logic could allow us to model the varying degrees and relationships of beliefs and cognitive alignment among individuals within a public across societal domains—such as the economy, health, and politics. Each individual's mental model could theoretically be represented as a fuzzy set, where each belief or perception is associated with a membership function μ(*x*) that quantifies the degree of truth or belief in that concept on a scale from 0 (completely false) to 1 (completely true). The collection of individual MMs forms a fuzzy set *MM*_population_, where each person's beliefs contribute to the overall societal mental model. The public mental model itself, PMM, is then constructed by aggregating these individual membership functions using fuzzy logic operations such as conjunction, disjunction, or averaging. This aggregation can be represented mathematically as follows:


$$\mu_{\text{PMM}}\left(x\right)=\max\left(\mu_{\text{MM}1}\left(x\right),\mu_{\text{MM}2}\left(x\right),\ldots ,\mu_{\text{MM}n}\left(x\right)\right)$$

or


$$\mu_{\text{PMM}}\left(x\right)=\frac{1}{n}\overset{n}{\underset{i=1}{\sum }}\mu_{i}\left(x\right)$$

where μ_MM_*i*__(*x*) represents the membership function of the i-th individual's mental model concerning belief x, and n is the total number of individuals contributing to the public mental model. This process creates a composite PMM that reflects the collective beliefs and perceptions of the population, with varying levels of confidence and alignment. This formalism provides a structured approach to representing individual and Public Mental Models. Each individual's MM can be described as a formal set or vector:


$${\text{MM}}_{i}=\left\{\left(B_{1},\mu_{\left(B_{1}\right)}\right),\left(B_{2},\mu_{\left(B_{2}\right)}\right),\ldots ,\left(B_{m},\mu_{\left(B_{m}\right)}\right)\right\}$$

where *B*_*j*_ denotes the j-th belief or cognitive element, and μ_*B*_*j*__ is the corresponding membership function indicating the degree of belief or acceptance of that element for the individual i. The PMM is then defined as an aggregate set:


$$\text{PMM}=\{\left(B_{1},\mu_{\left(B_{1}\right)}^{\text{PMM}}\right),\left(B_{2},\mu_{\left(B_{2}\right)}^{\text{PMM}}\right),\ldots ,\left(B_{m},\mu_{\left(B_{m}\right)}^{\text{PMM}}\right)$$

where $\mu_{\left(B_{j}\right)}^{\text{PMM}}$ represents the collective acceptance or belief level of the public regarding belief *B*_*j*_. This formal structure enables systematic analysis of the public's cognitive framework and the identification of dominant or conflicting beliefs within a society. The formation of PMMs can be guided by fuzzy rules and inference systems. These rules allow us to model the influence of various factors on public beliefs and behaviors. For example, a simple fuzzy rule might state:


$$\text{if}\mu_{\text{MM}i}\left(\text{“Trust in Government”}\right)>0.7\text{ then }\mu_{\text{PMM}}$$

(“Support for Policy X”)≈high.

Such rules help in understanding how individual beliefs converge to form a coherent public stance on specific issues. The application of fuzzy inference systems could help to simulate and predict the evolution of PMMs in response to changes in individual MMs or external stimuli. As the third step, feedback loops are used to continuously update the PMMF with the latest behavioral and contextual data to enable the digital twin representation of PMM to evolve in, to improve the performance of monitored CPSS, as public opinion, sentiment, or collective understanding shifts. To better clarify the PMMF novelty we explain how the data and its collection for PMMF differs from data collection for SA systems. The process of data collection and indicator identification in the Public Mental Model Framework (PMMF) differs from Situation Awareness (SA) in several essential ways due to their distinct goals and focuses. PMMF focuses on understanding and modeling Public Mental Models, which are shaped by perceptions, beliefs, emotions, and social influences. The data collected for PMMF often centers mainly on subjective, qualitative indicators such as public opinion, social media sentiments, cultural norms, and trust in institutions, particularly focused on psychological, sociological, and behavioral metrics. This might include data from surveys, interviews, social media analysis, or sentiment analysis. This data is collected to analyze long-term shifts in public perception and collective behavior. SA, on the other hand, is more concerned with real-time, operational data that can be used to make timely decisions in dynamic environments. SA focuses on objective, quantitative indicators like sensor data, live surveillance, or operational reports, which are necessary for understanding the current state of a situation in a crisis or changing scenario. SA data stems often from physical or technological (cyber) systems, such as traffic flow, resource availability, environmental sensors, and threat detection systems. The data collected is used to make immediate situational decisions, focusing on current status rather than long-term behavioral trends and patterns. In PMMF, indicators are often psychosocial and focus on understanding how external triggers (e.g., economic changes, political events) influence public behavior. These indicators can be abstract and require more narrative or context-based interpretation. For example, understanding the public's trust in governmental institutions during a health crisis would be a key indicator in PMMF. PMMF supports strategic decision-making by offering insights into long-term public behavior and sentiment trends. It can be used for planning interventions that shape public perception and behavior, often in response to societal challenges like misinformation or policy acceptance. In SA, indicators are more operational and objective—for instance, tracking resources, monitoring threats, and assessing infrastructure integrity. These indicators are directly tied to the immediate understanding of the physical environment or system status. SA aids tactical decision-making in real-time, helping operators and decision-makers quickly understand and react to immediate threats or events. For example, during a natural disaster, SA data would be used to coordinate emergency services, manage evacuations, and monitor environmental conditions. Regarding temporal view, PMMF looks at long-term data, aiming to capture changes in Public Mental Models over time. It is designed to understand slow-moving societal trends, shifts in public opinion, and the impact of various policies on public beliefs. SA in turn is focused on real-time or near-real-time data, ensuring that the latest situational information is available for decision-making to manage immediate crises or operational situations. Data collection and analysis in PMMF and SA serve different purposes in indicator identification. While PMMF is focused on understanding collective mental models and long-term societal changes through a blend of qualitative and quantitative data, SA is focused on immediate, real-time, and operationally relevant data to manage dynamic, fast-changing situations. These differing approaches are complementary and can support one another in complex environments like Cyber-Physical-Social Systems (CPSS). To finetune the PMMF and to influence the public behavior towards desired directions, dynamic interventions can be designed based on constructed PMMs. The PMMF incorporates two feedback loops for interventions in Public Behavior Patterns (PBP) and mental models: The first feedback loop: Flows from PBP to PMM and refines the PMMF, ensuring its accuracy by allowing interventions aimed at validating and modifying the PMM construct within the PMMF. As public behavior patterns evolve, the framework adjusts its representation of the respective PMMs. The second feedback loop: Flows from PMM to PBM to allow interventions in public behaviors. This loop is based on simulations of PBP in the virtual world as a part of the PMM construct, allowing for the testing the interventions that could influence real-world behaviors. Insights from this analysis can be used to design interventions that shape behavior in desired ways, such as promoting safety during a crisis or encouraging positive public health practices. Constructing digital models for particular PMMs, enables PMMF to be continuously updated and validated.

However, to ensure reliable analysis of PMM, PBP and prediction of possible emergent behavior, it is critical that suitable modelling and simulation approaches are selected. Most likely an interoperable combination or suite of (semi)formal modelling approaches will be necessary. This could for example include approaches from dynamic interaction-centered multi-agent modelling to static enterprise architecture frameworks and simulation tools that could be used to simulate potential future scenarios and predict public behavior based on evolving social, technological, and environmental factors. With this deeper comprehension in place, the framework can then forecast how public behaviors might evolve in response to future changes or interventions or how different events might influence public responses. This foresight would allow decision-makers to anticipate potential outcomes and craft proactive strategies. For instance, the system might predict how the public will respond to a new vaccination campaign, how changes in financial aid could influence social stability or how might public attitudes shift during an economic downturn? This approach helps stakeholders explore “what-if” scenarios and test strategies before implementation. A feedback loop underpins the entire framework, enabling constant monitoring of the situation as it unfolds. As new data flows in, the system refines its understanding of Public Mental Models and behavior patterns, ensuring that its assessments remain accurate and up to date. This dynamic adaptability allows for ongoing adjustments to interventions, ensuring they stay aligned with shifting public perceptions and emerging trends. By remaining responsive, the system helps ensure that interventions remain effective, even in fast-evolving situations.

## 7 Conclusion

Public Mental Models can be considered important for shaping public understanding, behavior patterns, and decision-making, especially during critical situations like the COVID-19 pandemic. Public Behavior Patterns, which in turn are influenced by social domains like cultural, medical, cyber, economic, and political domains could be better understood through Public Mental Models Framework. Inspired by neuroergonomics, the proposed Public Mental Models Framework aligns this understanding with motivation, opportunity, and ability i.e., MOA theory and together with analysis of societal indicators (markers, triggers and drivers) that influence peoples behavior. The aim is to provide a conceptual framework to bring a person back into Cyber-Physical-Social Systems research, especially complex systems with human-machine interactions that rely on AI, estimate the performance of these Systems and to support more effective critical situation management.

Public Mental Models Framework categorizes Mental Models at different societal levels—individual, groups/teams, institutional/organizational, community, and societal—highlighting how Shared Mental Models influence human behavior. In this categorization, the Public Mental Models exist at both the societal and community levels, Common Mental Models are shared among different groups of people working or doing something together and Crowd Mental Models are dynamic, reflecting collective behavior in temporary gatherings. Understanding these models helps anticipate behavior, manage crowds, and enhance public safety during crises.

In our earlier works, the role of Situation Awareness and Stress Management in Public Behavior Patterns have been identified as central to effective crisis management. High stress levels and cognitive overload are noted as significant factors that diminish Situation Awareness and impair decision-making. Current paper emphasizes that accurate Public Mental Models, along with continuous monitoring of Public Behavior Patterns and Situation Awareness, can help counteract the adverse effects of stress and enhance the public's response to critical situations. Paper also brings out the differentiation between Public Mental Models Framework and Situation Awareness in data collection and analysis. Public Mental Models Framework focuses on understanding longer-term tendencies of societal indicators such as public opinion, cultural trends, and trust in institutions etc. This contrasts with data collection and analysis for Situation Awareness, which mainly emphasizes real-time, quantitative data for surrounding environment for immediate and operational crisis management.

To achieve this, the proposed Public Mental Models Framework seeks to create conditions to design digital twin-type models for both Public Mental Models and also for Public Behavior Patterns using digital modeling that involves advanced AI algorithms and formal or semi-formal logic e.g., fuzzy logic combined with AI. This enables analysis of various societal indicators, shared beliefs and cognitive processes to forecast public responses in both in critical situations and prolonged crises. By enabling simulations for “what-if” scenarios, Public Mental Models Framework helps anticipate responses to events like health crises or economic changes, allowing proactive, strategic planning. However, we also recognize the complex interplay between Mental Models, Situation Awareness, Public Behavior Patterns and the intricate dynamics that influence collective understanding and actions. Challenges in Cyber-Physical-Social Systems include accurately predicting human behavior and integrating human-centric design. Technologies such as digital twinning and AI could enhance systems performance by simulating Public Mental Models for better decision-making, but many theoretical and technical challenges remain.

On the other hand, it could be appropriate to consider a Public Mental Models Framework as akin to a collection of information comprising verbally presented mental images, that is partially encapsulated within a metaphorical “book,” and this “book” never encompasses the entirety of truth (which is inherently transient and in constant flux)? The book-shaped Public Mental Models Framework could be pre-used, its contents acknowledged, and utilized in practical simulation experiments when constructing simulation scenarios. The evolution of the book's content should not pose a challenge (akin to a compilation of statutes and regulations to which a group of legal experts consistently appends new nuances and amendments). Familiarity with the Public Mental Models Framework would provide the simulation operator with vague guidelines on configuring a simulation experiment to investigate a pertinent process, grounded in the presently valid “statutes” (yet nothing more). However, the potential utility of this framework lies in its capacity to consult the Public Mental Models Framework for a comprehensive comprehension or to fabricate simulations associated with, for instance, climate change, or even as a loose guide. It could serve as a primer or prelude, imparting current insights without claiming to provide an exhaustive or ultimate treatment of the subject. The Public Mental Models Framework could constitute a perpetually evolving reservoir of knowledge regarding a specific topic, systematically structured akin to a book. It can serve as a cornerstone for diverse research undertakings and furnish directives for thorough explorations. Yet, it's acknowledged that it does not purport to embody an absolute or definitive truth concerning the subject. The main value of the proposed Public Mental Model Framework concept lies in its potential to guide interventions for positive behavior change by bringing a person back into Cyber-Physical-Social Systems for especially in coping with critical situations. Next steps could involve validating the Public Mental Models Framework (PMMF) to link societal indicators with observable Public Behavior Patterns. Computationally, PMMF could be implemented as a digital twin prototype using agent-based and fuzzy-logic simulations to test “what-if” crisis scenarios such as pandemic communication or AI-trust evolution.
